# *cis*-Bis[(1-adamantylmeth­yl)amine-κ*N*]­dichloridoplatinum(II) *N*,*N*-dimethyl­formamide solvate

**DOI:** 10.1107/S1600536809037982

**Published:** 2009-10-03

**Authors:** Fernande D. Rochon, Christian Tessier

**Affiliations:** a Département de Chimie, Université du Québec à Montréal, CP 8888, Succ. Centre-ville, Montréal, Québec, Canada H3*C* 3P8

## Abstract

The asymmetric unit of the title compound {systematic name: *cis*-dichloridobis[(3,7-dimethylbicyclo­[3.3.1]non-1-ylmeth­yl)­amine-κ*N*]platinum(II) *N*,*N*-dimethyl­formamide solvate}, [PtCl_2_(C_11_H_19_N)_2_]·C_3_H_7_NO, consists of two metrically similar Pt complexes and two dimethyl­formamide solvent mol­ecules. Each Pt^II^ center is coordinated by the amine groups of two (1-adamantylmeth­yl)amine ligands and two Cl atoms in a *cis*-square-planar arrangement. The Pt^II^ centers lie slightly outside [0.031 (4) and 0.038 (4) Å] the coordination planes. The N—Pt—N and Cl—Pt—Cl angles [92.1 (4)–92.30 (11)°] are slightly more open than the N—Pt—Cl angles [87.3 (3)–88.3 (3)°]. N—H⋯O and N—H⋯Cl inter­molecular hydrogen bonds are observed, forming two discrete pairs of complexes and solvent mol­ecules.

## Related literature

For the anti­viral and anti­tumor activity of Pt complexes with polycyclic cages such as adamantamine, see: Hay *et al.* (1985 ▶); Ho *et al.* (1972 ▶); Widell *et al.* (1986 ▶). The synthesis and spectroscopic characterization of the title compound is described by Rochon *et al.* (1993 ▶).
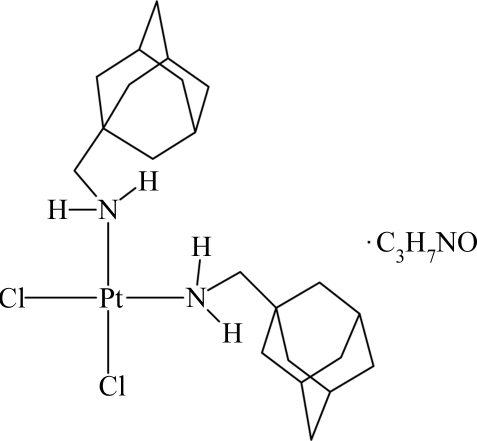


## Experimental

### 

#### Crystal data

[PtCl_2_(C_11_H_19_N)_2_]·C_3_H_7_NO
*M* *_r_* = 669.63
Triclinic, 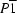

*a* = 12.299 (3) Å
*b* = 14.035 (4) Å
*c* = 15.644 (4) Å
α = 81.137 (3)°
β = 82.323 (3)°
γ = 89.292 (3)°
*V* = 2644.3 (12) Å^3^
*Z* = 4
Mo *K*α radiation
μ = 5.53 mm^−1^
*T* = 200 K
0.35 × 0.29 × 0.23 mm

#### Data collection

Bruker SMART APEXII CCD diffractometer
Absorption correction: multi-scan (*SADABS*; Bruker, 2001 ▶) *T*_min_ = 0.151, *T*_max_ = 0.281
26513 measured reflections
9285 independent reflections
5724 reflections with *I* > 2σ(*I*)
*R*_int_ = 0.099

#### Refinement

*R*[*F* ^2^ > 2σ(*F* ^2^)] = 0.059
*wR*(*F* ^2^) = 0.152
*S* = 0.97
9285 reflections
581 parameters
H-atom parameters constrained
Δρ_max_ = 7.80 e Å^−3^
Δρ_min_ = −2.82 e Å^−3^

### 

Data collection: *APEX2* (Bruker, 2007 ▶); cell refinement: *SAINT* (Bruker, 2007 ▶); data reduction: *SAINT*; program(s) used to solve structure: *SHELXS97* (Sheldrick, 2008 ▶); program(s) used to refine structure: *SHELXL97* (Sheldrick, 2008 ▶); molecular graphics: *SHELXTL* (Sheldrick, 2008 ▶); software used to prepare material for publication: *SHELXTL* and *publCIF* (Westrip, 2009 ▶).

## Supplementary Material

Crystal structure: contains datablocks I, global. DOI: 10.1107/S1600536809037982/hy2221sup1.cif

Structure factors: contains datablocks I. DOI: 10.1107/S1600536809037982/hy2221Isup2.hkl

Additional supplementary materials:  crystallographic information; 3D view; checkCIF report

## Figures and Tables

**Table 1:** Selected bond lengths (Å)

Pt1—N11	2.040 (9)
Pt1—N12	2.026 (9)
Pt1—Cl11	2.304 (3)
Pt1—Cl12	2.312 (3)
Pt2—N21	2.048 (8)
Pt2—N22	2.056 (9)
Pt2—Cl21	2.307 (3)
Pt2—Cl22	2.306 (3)

**Table 2:** Hydrogen-bond geometry (Å, °)

*D*—H⋯*A*	*D*—H	H⋯*A*	*D*⋯*A*	*D*—H⋯*A*
N11—H11*A*⋯O2	0.92	1.95	2.863 (13)	170
N11—H11*B*⋯Cl12^i^	0.92	2.64	3.409 (10)	141
N12—H12*A*⋯Cl11^i^	0.92	2.63	3.299 (9)	131
N12—H12*B*⋯O2	0.92	1.93	2.843 (11)	175
N21—H21*A*⋯Cl22^ii^	0.92	2.55	3.238 (10)	132
N21—H21*B*⋯O1	0.92	1.91	2.821 (12)	173
N22—H22*A*⋯O1	0.92	1.94	2.843 (11)	165
N22—H22*B*⋯Cl21^ii^	0.92	2.64	3.354 (10)	136

Symmetry codes: (i) 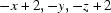; (ii) 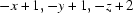.

**Fig. 1.**
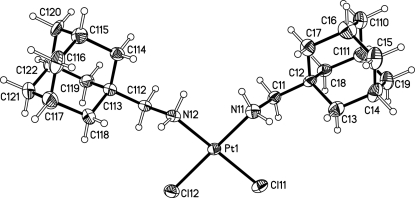
A view of one of the crystallographically independent molecules of the title compound. Displacement ellipsoids are shown at the 50% probability level.

**Fig. 2.**
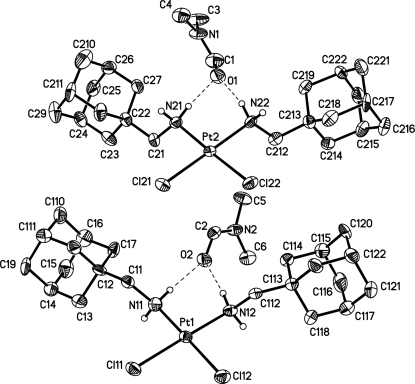
A view of the asymmetric unit of the title compound. Displacement ellipsoids are drawn at the 50% probability level. H atoms not participating in hydrogen bonds have been omitted for clarity.

**Fig. 3.**
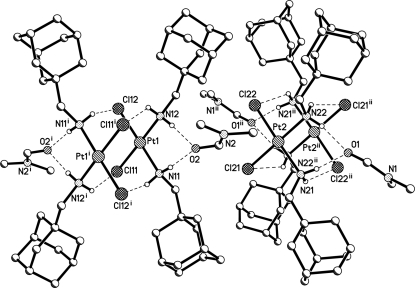
The hydrogen bonding pattern in the title compound. H atoms not participating in hydrogen bonds have been omitted for clarity. [Symmetry codes: (i) -x+2, -y, -z+2; (ii) -x+1, -y+1, -z+2.]

## supplementary crystallographic information

### Comment

*Cisplatin* [*cis*-Pt(NH_3_)_2_Cl_2_] is now a well known antitumor drug, but it has numerous side effects and resistance to the drug is an important problem. Replacement of the NH_3_ ligand by an amine more compatible to the human system might possibly surmount some of these problems. One such amine is the polycyclic cage molecule adamantanamine, which has been demonstrated to exhibit both antiviral (Hay *et al.*, 1985; Widell *et al.*, 1986) and antitumor activity (Ho *et al.*, 1972). The synthesis and the spectroscopic study of Pt^II^ compounds with adamantanamine derivatives have been reported (Rochon *et al.*, 1993).

In the title compound (one of the Pt complex is shown in Fig. 1), the Pt metal center exhibits a *cis* square-planar geometry formed by two amine groups from (1-adamantylmethyl)amine and two Cl atoms. The Pt centers are slightly outside [0.031 (4) and 0.038 (4) Å] the N_2_Cl_2_ planes. The bulky adamantane cycles slightly open the N—Pt—N angles [92.1 (4)°] compared to the N—Pt—Cl angles [87.3 (3)–88.3 (3)°]. However, the Cl—Pt—Cl angles are also opened to the same extent [92.17 (11)–92.30 (11)°]. The Pt—N [2.026 (9)–2.056 (9) Å] and Pt—Cl [2.304 (3)–2.312 (3) Å] are normal as well as all other bond distances and angles.

The asymmetric unit of the title compound is described as two crystallographically independent Pt complexes and two dimethylformamide (DMF) solvent molecules linked by N—H···O hydrogen bonds (Fig. 2) between the amine groups and the O atoms from the DMF molecules. N—H···Cl hydrogen bonds between the amine groups and Cl atoms from symmetry equivalent complexes are also observed, forming discrete pairs of complexes and solvent molecules (Fig. 3).

### Experimental

One mmol of K_2_[PtCl_4_] and 2 mmol of (1-adamantylmethyl)amine were heated in a DMF solution at 80°C for 3 h. The solution was concentrated, cooled to 0°C and the KCl was filtered off. The mixture was evaporated to dryness and the residue washed with ether, acetone and then with water. After drying, the residue was washed with ether and dried. Yield 68%, dec. 195–225°C, ν(Pt—Cl) IR 341, 325, Raman 334, 325 cm^-1^, ^195^Pt NMR -2213 p.p.m. The crystals were recrystalized in DMF for crystallographic studies.

### Refinement

H atoms were placed at calculated positions (C—H = 0.95–1.00 Å, N—H = 0.92 Å) and were allowed to ride on their parent atoms, with *U*_iso_(H) = 1.2*U*_eq_(C,N) for the CH, CH_2_ and NH_2_ groups and *U*_iso_(H) = 1.5*U*_eq_(C) for the CH_3_ groups. The highest residual electron density was found 1.0 Å from Pt2.

### Crystal data

**Table:**

[PtCl_2_(C_11_H_19_N)_2_]·C_3_H_7_NO	*Z* = 4
*M**_r_* = 669.63	*F*(000) = 1344
Triclinic, *P*1	*D*_x_ = 1.682 Mg m^−^^3^
Hall symbol: -P 1	Mo *K*α radiation, λ = 0.71073 Å
*a* = 12.299 (3) Å	Cell parameters from 6035 reflections
*b* = 14.035 (4) Å	θ = 2.2–26.3°
*c* = 15.644 (4) Å	µ = 5.53 mm^−^^1^
α = 81.137 (3)°	*T* = 200 K
β = 82.323 (3)°	Block, yellow
γ = 89.292 (3)°	0.35 × 0.29 × 0.23 mm
*V* = 2644.3 (12) Å^3^	

### Data collection

**Table:**

Bruker SMART APEXII CCD diffractometer	9285 independent reflections
Radiation source: normal-focus sealed tube	5724 reflections with *I* > 2σ(*I*)
graphite	*R*_int_ = 0.099
φ and ω scans	θ_max_ = 25.0°, θ_min_ = 1.5°
Absorption correction: multi-scan (*SADABS*; Bruker, 2001)	*h* = −14→14
*T*_min_ = 0.151, *T*_max_ = 0.281	*k* = −16→16
26513 measured reflections	*l* = −18→18

### Refinement

**Table:**

Refinement on *F*^2^	Primary atom site location: structure-invariant direct methods
Least-squares matrix: full	Secondary atom site location: difference Fourier map
*R*[*F*^2^ > 2σ(*F*^2^)] = 0.059	Hydrogen site location: inferred from neighbouring sites
*wR*(*F*^2^) = 0.152	H-atom parameters constrained
*S* = 0.97	*w* = 1/[σ^2^(*F*_o_^2^) + (0.0732*P*)^2^]
9285 reflections	(Δ/σ)_max_ = 0.001
581 parameters	Δρ_max_ = 7.80 e Å^−^^3^
0 restraints	Δρ_min_ = −2.82 e Å^−^^3^
0 constraints	

### Fractional atomic coordinates and isotropic or equivalent isotropic displacement parameters (Å^2^)

**Table:**

	*x*	*y*	*z*	*U*_iso_*/*U*_eq_	
Pt1	1.00670 (3)	0.12043 (3)	0.99942 (3)	0.02177 (15)	
Pt2	0.50836 (3)	0.38164 (3)	0.99668 (3)	0.02119 (15)	
Cl11	1.1120 (2)	0.0882 (2)	1.1116 (2)	0.0311 (7)	
Cl12	1.1596 (2)	0.1589 (2)	0.8970 (2)	0.0313 (7)	
Cl21	0.6257 (2)	0.3401 (2)	1.0995 (2)	0.0306 (7)	
Cl22	0.6520 (2)	0.4151 (2)	0.8854 (2)	0.0308 (7)	
N11	0.8725 (7)	0.0841 (7)	1.0899 (6)	0.028 (2)	
H11A	0.8125	0.0835	1.0606	0.034*	
H11B	0.8818	0.0220	1.1168	0.034*	
N12	0.9139 (7)	0.1428 (6)	0.9006 (6)	0.024 (2)	
H12A	0.9334	0.0971	0.8651	0.029*	
H12B	0.8421	0.1302	0.9247	0.029*	
N21	0.3800 (7)	0.3594 (7)	1.0955 (6)	0.025 (2)	
H21A	0.3861	0.4046	1.1315	0.030*	
H21B	0.3165	0.3728	1.0710	0.030*	
N22	0.4049 (7)	0.4215 (7)	0.9047 (6)	0.025 (2)	
H22A	0.3353	0.4261	0.9336	0.030*	
H22B	0.4255	0.4822	0.8764	0.030*	
C11	0.8455 (9)	0.1455 (8)	1.1597 (8)	0.026 (3)	
H11C	0.8083	0.2042	1.1347	0.031*	
H11D	0.9151	0.1664	1.1767	0.031*	
C12	0.7745 (8)	0.0994 (8)	1.2407 (8)	0.027 (3)	
C13	0.8333 (10)	0.0124 (9)	1.2863 (9)	0.037 (3)	
H13A	0.8459	−0.0371	1.2474	0.044*	
H13B	0.9056	0.0332	1.2985	0.044*	
C14	0.7644 (11)	−0.0307 (9)	1.3717 (9)	0.040 (3)	
H14	0.8035	−0.0870	1.4008	0.048*	
C15	0.6534 (11)	−0.0633 (10)	1.3540 (10)	0.046 (4)	
H15A	0.6093	−0.0920	1.4095	0.055*	
H15B	0.6633	−0.1130	1.3151	0.055*	
C16	0.5936 (10)	0.0243 (10)	1.3106 (9)	0.042 (4)	
H16	0.5208	0.0030	1.2983	0.050*	
C17	0.6638 (9)	0.0671 (9)	1.2227 (8)	0.033 (3)	
H17A	0.6739	0.0177	1.1835	0.040*	
H17B	0.6254	0.1227	1.1934	0.040*	
C18	0.7575 (9)	0.1732 (9)	1.3017 (8)	0.029 (3)	
H18A	0.7190	0.2295	1.2737	0.035*	
H18B	0.8299	0.1957	1.3122	0.035*	
C19	0.7469 (11)	0.0470 (10)	1.4320 (8)	0.042 (3)	
H19A	0.7021	0.0198	1.4875	0.050*	
H19B	0.8188	0.0670	1.4455	0.050*	
C110	0.5759 (10)	0.1016 (10)	1.3705 (9)	0.042 (3)	
H11E	0.5299	0.0753	1.4257	0.051*	
H11F	0.5382	0.1579	1.3417	0.051*	
C111	0.6906 (10)	0.1326 (10)	1.3894 (9)	0.037 (3)	
H111	0.6818	0.1832	1.4284	0.044*	
C112	0.9164 (8)	0.2395 (8)	0.8425 (7)	0.025 (3)	
H11G	0.9935	0.2625	0.8288	0.030*	
H11H	0.8749	0.2860	0.8755	0.030*	
C113	0.8697 (9)	0.2402 (8)	0.7573 (8)	0.028 (3)	
C114	0.7502 (8)	0.2058 (8)	0.7745 (8)	0.026 (3)	
H11I	0.7063	0.2472	0.8117	0.031*	
H11J	0.7457	0.1387	0.8057	0.031*	
C115	0.7041 (10)	0.2106 (9)	0.6873 (9)	0.033 (3)	
H115	0.6259	0.1881	0.6991	0.040*	
C116	0.7692 (11)	0.1466 (10)	0.6313 (9)	0.046 (4)	
H11K	0.7388	0.1496	0.5755	0.055*	
H11L	0.7637	0.0791	0.6613	0.055*	
C117	0.8906 (11)	0.1789 (10)	0.6129 (9)	0.046 (4)	
H117	0.9343	0.1358	0.5763	0.055*	
C118	0.9350 (9)	0.1754 (9)	0.7006 (8)	0.034 (3)	
H11M	0.9313	0.1082	0.7316	0.040*	
H11N	1.0129	0.1961	0.6898	0.040*	
C119	0.8761 (9)	0.3426 (9)	0.7076 (8)	0.033 (3)	
H11O	0.8344	0.3862	0.7438	0.040*	
H11P	0.9536	0.3647	0.6960	0.040*	
C120	0.7092 (10)	0.3150 (9)	0.6396 (9)	0.039 (3)	
H12C	0.6655	0.3573	0.6760	0.046*	
H12D	0.6785	0.3185	0.5838	0.046*	
C121	0.8971 (10)	0.2848 (10)	0.5652 (9)	0.042 (3)	
H12E	0.9745	0.3072	0.5535	0.051*	
H12F	0.8683	0.2886	0.5087	0.051*	
C122	0.8302 (10)	0.3481 (9)	0.6220 (9)	0.036 (3)	
H122	0.8347	0.4163	0.5916	0.043*	
C21	0.3643 (9)	0.2604 (8)	1.1537 (7)	0.025 (3)	
H21C	0.3335	0.2147	1.1208	0.030*	
H21D	0.4369	0.2359	1.1672	0.030*	
C22	0.2910 (9)	0.2626 (8)	1.2367 (8)	0.027 (3)	
C23	0.2776 (9)	0.1592 (8)	1.2867 (9)	0.033 (3)	
H23A	0.2448	0.1177	1.2510	0.040*	
H23B	0.3506	0.1330	1.2973	0.040*	
C24	0.2043 (10)	0.1579 (10)	1.3739 (9)	0.036 (3)	
H24	0.1973	0.0902	1.4055	0.043*	
C25	0.0903 (10)	0.1947 (9)	1.3549 (9)	0.041 (3)	
H25A	0.0592	0.1529	1.3185	0.049*	
H25B	0.0403	0.1921	1.4103	0.049*	
C26	0.0996 (10)	0.2980 (9)	1.3072 (9)	0.036 (3)	
H26	0.0254	0.3223	1.2955	0.044*	
C27	0.1764 (9)	0.3021 (9)	1.2207 (8)	0.034 (3)	
H27A	0.1837	0.3696	1.1908	0.041*	
H27B	0.1444	0.2634	1.1823	0.041*	
C28	0.3380 (10)	0.3259 (9)	1.2930 (8)	0.037 (3)	
H28A	0.3457	0.3928	1.2615	0.045*	
H28B	0.4119	0.3027	1.3040	0.045*	
C29	0.2507 (12)	0.2204 (10)	1.4295 (9)	0.050 (4)	
H29A	0.3228	0.1952	1.4437	0.059*	
H29B	0.2010	0.2195	1.4849	0.059*	
C210	0.1496 (14)	0.3623 (11)	1.3623 (11)	0.061 (5)	
H21E	0.1013	0.3623	1.4183	0.073*	
H21F	0.1558	0.4293	1.3311	0.073*	
C211	0.2644 (12)	0.3252 (10)	1.3804 (8)	0.042 (3)	
H211	0.2971	0.3670	1.4168	0.050*	
C212	0.3992 (9)	0.3588 (9)	0.8380 (8)	0.031 (3)	
H21G	0.3533	0.3019	0.8650	0.037*	
H21H	0.4741	0.3354	0.8211	0.037*	
C213	0.3545 (8)	0.4031 (8)	0.7564 (8)	0.026 (3)	
C214	0.3578 (9)	0.3252 (9)	0.6998 (8)	0.031 (3)	
H21I	0.3122	0.2697	0.7310	0.037*	
H21J	0.4341	0.3028	0.6879	0.037*	
C215	0.3149 (10)	0.3628 (9)	0.6132 (8)	0.032 (3)	
H215	0.3187	0.3102	0.5765	0.039*	
C216	0.3852 (10)	0.4487 (9)	0.5644 (8)	0.037 (3)	
H21K	0.4624	0.4286	0.5519	0.044*	
H21L	0.3582	0.4728	0.5082	0.044*	
C217	0.3781 (10)	0.5276 (9)	0.6210 (8)	0.035 (3)	
H217	0.4234	0.5841	0.5896	0.042*	
C218	0.4229 (9)	0.4896 (9)	0.7074 (8)	0.032 (3)	
H21M	0.5004	0.4703	0.6949	0.038*	
H21N	0.4201	0.5413	0.7441	0.038*	
C219	0.2345 (9)	0.4343 (9)	0.7761 (8)	0.030 (3)	
H21O	0.2301	0.4862	0.8127	0.036*	
H21P	0.1894	0.3790	0.8085	0.036*	
C220	0.1947 (9)	0.3931 (10)	0.6334 (8)	0.037 (3)	
H22C	0.1645	0.4156	0.5783	0.045*	
H22D	0.1503	0.3372	0.6654	0.045*	
C221	0.2589 (11)	0.5595 (10)	0.6414 (9)	0.044 (4)	
H22E	0.2552	0.6098	0.6796	0.053*	
H22F	0.2302	0.5868	0.5867	0.053*	
C222	0.1897 (10)	0.4717 (10)	0.6873 (8)	0.036 (3)	
H222	0.1116	0.4919	0.6998	0.044*	
O1	0.1953 (6)	0.4012 (6)	1.0075 (6)	0.038 (2)	
N1	0.0234 (8)	0.4174 (7)	1.0768 (8)	0.039 (3)	
C1	0.0970 (10)	0.3779 (10)	1.0247 (10)	0.045 (4)	
H1	0.0729	0.3271	0.9978	0.054*	
C3	−0.0896 (10)	0.3810 (10)	1.0928 (12)	0.059 (5)	
H3A	−0.0946	0.3247	1.0633	0.088*	
H3B	−0.1385	0.4315	1.0701	0.088*	
H3C	−0.1112	0.3622	1.1557	0.088*	
C4	0.0489 (10)	0.4994 (9)	1.1196 (10)	0.043 (3)	
H4A	0.1243	0.5217	1.0982	0.064*	
H4B	0.0416	0.4789	1.1829	0.064*	
H4C	−0.0021	0.5521	1.1062	0.064*	
O2	0.6969 (6)	0.1013 (6)	0.9856 (6)	0.035 (2)	
N2	0.5460 (7)	0.0836 (7)	0.9195 (7)	0.030 (2)	
C2	0.6048 (9)	0.1270 (9)	0.9700 (8)	0.029 (3)	
H2	0.5732	0.1803	0.9948	0.035*	
C5	0.4361 (9)	0.1158 (9)	0.9092 (10)	0.041 (4)	
H5A	0.4165	0.1666	0.9448	0.061*	
H5B	0.3847	0.0615	0.9281	0.061*	
H5C	0.4323	0.1412	0.8477	0.061*	
C6	0.5893 (9)	0.0038 (8)	0.8775 (9)	0.036 (3)	
H6A	0.6606	−0.0147	0.8962	0.055*	
H6B	0.5984	0.0231	0.8141	0.055*	
H6C	0.5384	−0.0511	0.8939	0.055*	

### Atomic displacement parameters (Å^2^)

**Table:**

	*U*^11^	*U*^22^	*U*^33^	*U*^12^	*U*^13^	*U*^23^
Pt1	0.0202 (2)	0.0212 (3)	0.0250 (3)	0.00066 (18)	−0.00497 (19)	−0.0053 (2)
Pt2	0.0207 (2)	0.0200 (3)	0.0230 (3)	0.00236 (18)	−0.00192 (19)	−0.0046 (2)
Cl11	0.0281 (15)	0.0326 (18)	0.0365 (19)	0.0051 (12)	−0.0141 (13)	−0.0093 (15)
Cl12	0.0241 (14)	0.0338 (18)	0.0350 (19)	0.0010 (12)	−0.0014 (12)	−0.0045 (14)
Cl21	0.0255 (14)	0.0323 (18)	0.0346 (19)	0.0047 (12)	−0.0092 (13)	−0.0031 (14)
Cl22	0.0259 (14)	0.0320 (18)	0.0324 (18)	0.0024 (12)	0.0055 (12)	−0.0066 (14)
N11	0.028 (5)	0.028 (6)	0.030 (6)	−0.001 (4)	−0.006 (4)	−0.008 (5)
N12	0.019 (5)	0.021 (5)	0.031 (6)	0.000 (4)	0.000 (4)	−0.003 (4)
N21	0.026 (5)	0.024 (6)	0.024 (6)	0.004 (4)	0.001 (4)	−0.002 (4)
N22	0.022 (5)	0.032 (6)	0.022 (6)	0.002 (4)	0.003 (4)	−0.007 (5)
C11	0.028 (6)	0.024 (7)	0.026 (7)	0.004 (5)	−0.006 (5)	−0.003 (5)
C12	0.024 (6)	0.023 (7)	0.032 (8)	0.008 (5)	−0.005 (5)	0.003 (5)
C13	0.039 (7)	0.034 (8)	0.037 (8)	0.010 (6)	−0.006 (6)	−0.002 (6)
C14	0.063 (9)	0.023 (7)	0.033 (8)	0.019 (6)	−0.009 (7)	−0.003 (6)
C15	0.064 (10)	0.036 (9)	0.036 (9)	−0.005 (7)	−0.005 (7)	−0.001 (7)
C16	0.041 (8)	0.040 (9)	0.046 (9)	−0.010 (6)	−0.006 (7)	−0.011 (7)
C17	0.038 (7)	0.040 (8)	0.024 (7)	−0.003 (6)	−0.006 (6)	−0.014 (6)
C18	0.025 (6)	0.033 (7)	0.032 (8)	0.000 (5)	−0.008 (5)	−0.007 (6)
C19	0.050 (8)	0.060 (10)	0.016 (7)	0.003 (7)	−0.005 (6)	−0.011 (7)
C110	0.044 (8)	0.056 (10)	0.026 (8)	0.000 (7)	−0.004 (6)	−0.003 (7)
C111	0.038 (7)	0.035 (8)	0.038 (9)	0.008 (6)	−0.007 (6)	−0.008 (6)
C112	0.019 (5)	0.030 (7)	0.026 (7)	0.006 (5)	0.000 (5)	−0.005 (5)
C113	0.023 (6)	0.033 (7)	0.026 (7)	0.004 (5)	−0.001 (5)	−0.003 (6)
C114	0.022 (6)	0.029 (7)	0.028 (7)	−0.002 (5)	−0.002 (5)	−0.007 (5)
C115	0.034 (7)	0.034 (8)	0.035 (8)	0.000 (5)	−0.009 (6)	−0.012 (6)
C116	0.066 (10)	0.046 (9)	0.031 (9)	−0.002 (7)	−0.018 (7)	−0.013 (7)
C117	0.051 (8)	0.054 (10)	0.033 (9)	0.021 (7)	−0.003 (6)	−0.012 (7)
C118	0.033 (7)	0.041 (8)	0.025 (7)	0.004 (6)	0.002 (5)	−0.003 (6)
C119	0.023 (6)	0.038 (8)	0.033 (8)	0.001 (5)	−0.003 (5)	0.007 (6)
C120	0.044 (8)	0.049 (9)	0.026 (8)	0.016 (6)	−0.018 (6)	−0.002 (6)
C121	0.042 (7)	0.059 (10)	0.023 (8)	0.009 (6)	−0.002 (6)	0.000 (7)
C122	0.038 (7)	0.030 (8)	0.035 (8)	0.005 (6)	−0.003 (6)	0.005 (6)
C21	0.028 (6)	0.026 (7)	0.022 (7)	−0.003 (5)	−0.008 (5)	−0.001 (5)
C22	0.030 (6)	0.024 (7)	0.027 (7)	−0.004 (5)	−0.009 (5)	−0.002 (5)
C23	0.031 (6)	0.024 (7)	0.046 (9)	−0.005 (5)	−0.020 (6)	0.001 (6)
C24	0.038 (7)	0.035 (8)	0.032 (8)	−0.005 (6)	−0.008 (6)	0.008 (6)
C25	0.048 (8)	0.039 (8)	0.028 (8)	−0.006 (6)	0.010 (6)	0.004 (6)
C26	0.039 (7)	0.032 (8)	0.036 (8)	0.004 (6)	0.002 (6)	−0.002 (6)
C27	0.032 (7)	0.043 (8)	0.025 (7)	0.004 (6)	−0.005 (5)	0.001 (6)
C28	0.053 (8)	0.032 (8)	0.029 (8)	−0.011 (6)	−0.010 (6)	−0.009 (6)
C29	0.072 (10)	0.047 (9)	0.030 (9)	−0.017 (8)	−0.017 (7)	0.002 (7)
C210	0.100 (13)	0.033 (9)	0.043 (10)	−0.003 (8)	0.015 (9)	−0.003 (8)
C211	0.067 (9)	0.042 (9)	0.017 (7)	−0.010 (7)	−0.003 (6)	−0.008 (6)
C212	0.029 (6)	0.032 (7)	0.031 (8)	0.001 (5)	−0.002 (5)	−0.009 (6)
C213	0.022 (6)	0.031 (7)	0.027 (7)	−0.003 (5)	−0.006 (5)	−0.002 (6)
C214	0.019 (6)	0.042 (8)	0.032 (8)	0.003 (5)	−0.004 (5)	−0.006 (6)
C215	0.039 (7)	0.033 (8)	0.028 (8)	−0.011 (6)	−0.001 (6)	−0.015 (6)
C216	0.038 (7)	0.051 (9)	0.020 (7)	−0.010 (6)	0.003 (5)	−0.006 (6)
C217	0.047 (8)	0.044 (8)	0.010 (7)	−0.009 (6)	0.003 (5)	0.004 (6)
C218	0.031 (6)	0.030 (7)	0.037 (8)	−0.012 (5)	0.001 (5)	−0.016 (6)
C219	0.025 (6)	0.042 (8)	0.026 (7)	−0.003 (5)	0.001 (5)	−0.013 (6)
C220	0.032 (7)	0.055 (9)	0.025 (8)	−0.002 (6)	−0.009 (5)	−0.001 (7)
C221	0.063 (9)	0.039 (9)	0.031 (8)	0.009 (7)	−0.015 (7)	0.001 (7)
C222	0.032 (7)	0.050 (9)	0.027 (8)	0.015 (6)	−0.010 (6)	−0.001 (7)
O1	0.022 (4)	0.051 (6)	0.038 (6)	0.001 (4)	−0.001 (4)	0.002 (4)
N1	0.037 (6)	0.023 (6)	0.060 (8)	0.007 (5)	−0.009 (5)	−0.015 (6)
C1	0.042 (8)	0.035 (8)	0.060 (11)	0.000 (6)	−0.009 (7)	−0.015 (7)
C3	0.024 (7)	0.040 (9)	0.113 (15)	0.002 (6)	0.002 (8)	−0.026 (9)
C4	0.045 (8)	0.031 (8)	0.057 (10)	0.000 (6)	−0.012 (7)	−0.017 (7)
O2	0.021 (4)	0.047 (6)	0.037 (6)	−0.001 (4)	−0.007 (4)	−0.002 (4)
N2	0.022 (5)	0.026 (6)	0.044 (7)	0.001 (4)	−0.004 (4)	−0.013 (5)
C2	0.031 (7)	0.028 (7)	0.029 (8)	0.001 (5)	0.001 (5)	−0.008 (6)
C5	0.024 (6)	0.035 (8)	0.062 (10)	0.000 (5)	−0.010 (6)	−0.002 (7)
C6	0.036 (7)	0.026 (7)	0.048 (9)	−0.004 (5)	−0.004 (6)	−0.010 (6)

### Geometric parameters (Å, °)

**Table:**

Pt1—N11	2.040 (9)	C21—H21C	0.9900
Pt1—N12	2.026 (9)	C21—H21D	0.9900
Pt1—Cl11	2.304 (3)	C22—C28	1.512 (16)
Pt1—Cl12	2.312 (3)	C22—C23	1.539 (16)
Pt2—N21	2.048 (8)	C22—C27	1.545 (15)
Pt2—N22	2.056 (9)	C23—C24	1.529 (17)
Pt2—Cl21	2.307 (3)	C23—H23A	0.9900
Pt2—Cl22	2.306 (3)	C23—H23B	0.9900
N11—C11	1.493 (14)	C24—C29	1.494 (18)
N11—H11A	0.9200	C24—C25	1.539 (17)
N11—H11B	0.9200	C24—H24	1.0000
N12—C112	1.511 (14)	C25—C26	1.524 (17)
N12—H12A	0.9200	C25—H25A	0.9900
N12—H12B	0.9200	C25—H25B	0.9900
N21—C21	1.538 (14)	C26—C210	1.53 (2)
N21—H21A	0.9200	C26—C27	1.537 (16)
N21—H21B	0.9200	C26—H26	1.0000
N22—C212	1.474 (15)	C27—H27A	0.9900
N22—H22A	0.9200	C27—H27B	0.9900
N22—H22B	0.9200	C28—C211	1.535 (17)
C11—C12	1.501 (15)	C28—H28A	0.9900
C11—H11C	0.9900	C28—H28B	0.9900
C11—H11D	0.9900	C29—C211	1.551 (18)
C12—C18	1.509 (16)	C29—H29A	0.9900
C12—C17	1.515 (15)	C29—H29B	0.9900
C12—C13	1.540 (16)	C210—C211	1.55 (2)
C13—C14	1.526 (17)	C210—H21E	0.9900
C13—H13A	0.9900	C210—H21F	0.9900
C13—H13B	0.9900	C211—H211	1.0000
C14—C15	1.518 (18)	C212—C213	1.501 (16)
C14—C19	1.544 (17)	C212—H21G	0.9900
C14—H14	1.0000	C212—H21H	0.9900
C15—C16	1.540 (19)	C213—C214	1.506 (16)
C15—H15A	0.9900	C213—C218	1.528 (15)
C15—H15B	0.9900	C213—C219	1.540 (14)
C16—C110	1.534 (18)	C214—C215	1.535 (17)
C16—C17	1.561 (17)	C214—H21I	0.9900
C16—H16	1.0000	C214—H21J	0.9900
C17—H17A	0.9900	C215—C216	1.534 (16)
C17—H17B	0.9900	C215—C220	1.538 (16)
C18—C111	1.535 (17)	C215—H215	1.0000
C18—H18A	0.9900	C216—C217	1.515 (17)
C18—H18B	0.9900	C216—H21K	0.9900
C19—C111	1.491 (18)	C216—H21L	0.9900
C19—H19A	0.9900	C217—C221	1.534 (17)
C19—H19B	0.9900	C217—C218	1.545 (17)
C110—C111	1.560 (17)	C217—H217	1.0000
C110—H11E	0.9900	C218—H21M	0.9900
C110—H11F	0.9900	C218—H21N	0.9900
C111—H111	1.0000	C219—C222	1.575 (16)
C112—C113	1.519 (16)	C219—H21O	0.9900
C112—H11G	0.9900	C219—H21P	0.9900
C112—H11H	0.9900	C220—C222	1.484 (18)
C113—C118	1.517 (16)	C220—H22C	0.9900
C113—C119	1.523 (16)	C220—H22D	0.9900
C113—C114	1.528 (14)	C221—C222	1.532 (17)
C114—C115	1.538 (16)	C221—H22E	0.9900
C114—H11I	0.9900	C221—H22F	0.9900
C114—H11J	0.9900	C222—H222	1.0000
C115—C116	1.501 (17)	O1—C1	1.239 (14)
C115—C120	1.536 (17)	N1—C1	1.312 (16)
C115—H115	1.0000	N1—C3	1.463 (15)
C116—C117	1.542 (18)	N1—C4	1.474 (15)
C116—H11K	0.9900	C1—H1	0.9500
C116—H11L	0.9900	C3—H3A	0.9800
C117—C118	1.536 (18)	C3—H3B	0.9800
C117—C121	1.555 (19)	C3—H3C	0.9800
C117—H117	1.0000	C4—H4A	0.9800
C118—H11M	0.9900	C4—H4B	0.9800
C118—H11N	0.9900	C4—H4C	0.9800
C119—C122	1.512 (18)	O2—C2	1.229 (13)
C119—H11O	0.9900	N2—C2	1.354 (14)
C119—H11P	0.9900	N2—C5	1.441 (14)
C120—C122	1.541 (16)	N2—C6	1.445 (14)
C120—H12C	0.9900	C2—H2	0.9500
C120—H12D	0.9900	C5—H5A	0.9800
C121—C122	1.513 (17)	C5—H5B	0.9800
C121—H12E	0.9900	C5—H5C	0.9800
C121—H12F	0.9900	C6—H6A	0.9800
C122—H122	1.0000	C6—H6B	0.9800
C21—C22	1.483 (15)	C6—H6C	0.9800
			
N12—Pt1—N11	92.1 (4)	C22—C21—N21	113.5 (9)
N12—Pt1—Cl11	177.6 (3)	C22—C21—H21C	108.9
N11—Pt1—Cl11	87.5 (3)	N21—C21—H21C	108.9
N12—Pt1—Cl12	88.2 (3)	C22—C21—H21D	108.9
N11—Pt1—Cl12	179.0 (3)	N21—C21—H21D	108.9
Cl11—Pt1—Cl12	92.17 (11)	H21C—C21—H21D	107.7
N21—Pt2—N22	92.1 (4)	C21—C22—C28	111.4 (9)
N21—Pt2—Cl22	177.0 (3)	C21—C22—C23	108.5 (10)
N22—Pt2—Cl22	87.3 (3)	C28—C22—C23	109.0 (10)
N21—Pt2—Cl21	88.3 (3)	C21—C22—C27	111.8 (10)
N22—Pt2—Cl21	178.8 (3)	C28—C22—C27	107.4 (10)
Cl22—Pt2—Cl21	92.30 (11)	C23—C22—C27	108.7 (9)
C11—N11—Pt1	118.1 (7)	C24—C23—C22	110.6 (10)
C11—N11—H11A	107.8	C24—C23—H23A	109.5
Pt1—N11—H11A	107.8	C22—C23—H23A	109.5
C11—N11—H11B	107.8	C24—C23—H23B	109.5
Pt1—N11—H11B	107.8	C22—C23—H23B	109.5
H11A—N11—H11B	107.1	H23A—C23—H23B	108.1
C112—N12—Pt1	120.2 (7)	C29—C24—C23	111.2 (10)
C112—N12—H12A	107.3	C29—C24—C25	109.7 (12)
Pt1—N12—H12A	107.3	C23—C24—C25	108.2 (10)
C112—N12—H12B	107.3	C29—C24—H24	109.2
Pt1—N12—H12B	107.3	C23—C24—H24	109.2
H12A—N12—H12B	106.9	C25—C24—H24	109.2
C21—N21—Pt2	119.8 (7)	C26—C25—C24	109.8 (10)
C21—N21—H21A	107.4	C26—C25—H25A	109.7
Pt2—N21—H21A	107.4	C24—C25—H25A	109.7
C21—N21—H21B	107.4	C26—C25—H25B	109.7
Pt2—N21—H21B	107.4	C24—C25—H25B	109.7
H21A—N21—H21B	106.9	H25A—C25—H25B	108.2
C212—N22—Pt2	117.7 (7)	C25—C26—C210	109.8 (12)
C212—N22—H22A	107.9	C25—C26—C27	109.8 (10)
Pt2—N22—H22A	107.9	C210—C26—C27	107.5 (11)
C212—N22—H22B	107.9	C25—C26—H26	109.9
Pt2—N22—H22B	107.9	C210—C26—H26	109.9
H22A—N22—H22B	107.2	C27—C26—H26	109.9
N11—C11—C12	116.0 (9)	C26—C27—C22	111.0 (10)
N11—C11—H11C	108.3	C26—C27—H27A	109.4
C12—C11—H11C	108.3	C22—C27—H27A	109.4
N11—C11—H11D	108.3	C26—C27—H27B	109.4
C12—C11—H11D	108.3	C22—C27—H27B	109.4
H11C—C11—H11D	107.4	H27A—C27—H27B	108.0
C11—C12—C18	107.2 (9)	C22—C28—C211	111.4 (10)
C11—C12—C17	112.9 (10)	C22—C28—H28A	109.3
C18—C12—C17	109.1 (9)	C211—C28—H28A	109.3
C11—C12—C13	110.3 (9)	C22—C28—H28B	109.3
C18—C12—C13	107.9 (10)	C211—C28—H28B	109.3
C17—C12—C13	109.3 (10)	H28A—C28—H28B	108.0
C14—C13—C12	110.3 (10)	C24—C29—C211	109.8 (11)
C14—C13—H13A	109.6	C24—C29—H29A	109.7
C12—C13—H13A	109.6	C211—C29—H29A	109.7
C14—C13—H13B	109.6	C24—C29—H29B	109.7
C12—C13—H13B	109.6	C211—C29—H29B	109.7
H13A—C13—H13B	108.1	H29A—C29—H29B	108.2
C15—C14—C13	110.2 (12)	C26—C210—C211	110.1 (12)
C15—C14—C19	109.0 (11)	C26—C210—H21E	109.6
C13—C14—C19	108.9 (11)	C211—C210—H21E	109.6
C15—C14—H14	109.6	C26—C210—H21F	109.6
C13—C14—H14	109.6	C211—C210—H21F	109.6
C19—C14—H14	109.6	H21E—C210—H21F	108.2
C14—C15—C16	108.9 (11)	C28—C211—C210	108.8 (11)
C14—C15—H15A	109.9	C28—C211—C29	109.6 (11)
C16—C15—H15A	109.9	C210—C211—C29	107.8 (11)
C14—C15—H15B	109.9	C28—C211—H211	110.2
C16—C15—H15B	109.9	C210—C211—H211	110.2
H15A—C15—H15B	108.3	C29—C211—H211	110.2
C110—C16—C15	110.8 (12)	N22—C212—C213	116.6 (10)
C110—C16—C17	109.7 (10)	N22—C212—H21G	108.1
C15—C16—C17	108.9 (11)	C213—C212—H21G	108.1
C110—C16—H16	109.1	N22—C212—H21H	108.1
C15—C16—H16	109.1	C213—C212—H21H	108.1
C17—C16—H16	109.1	H21G—C212—H21H	107.3
C12—C17—C16	109.5 (10)	C212—C213—C214	106.1 (10)
C12—C17—H17A	109.8	C212—C213—C218	112.3 (9)
C16—C17—H17A	109.8	C214—C213—C218	109.4 (10)
C12—C17—H17B	109.8	C212—C213—C219	111.5 (10)
C16—C17—H17B	109.8	C214—C213—C219	108.3 (9)
H17A—C17—H17B	108.2	C218—C213—C219	109.2 (10)
C12—C18—C111	112.2 (10)	C213—C214—C215	110.8 (10)
C12—C18—H18A	109.2	C213—C214—H21I	109.5
C111—C18—H18A	109.2	C215—C214—H21I	109.5
C12—C18—H18B	109.2	C213—C214—H21J	109.5
C111—C18—H18B	109.2	C215—C214—H21J	109.5
H18A—C18—H18B	107.9	H21I—C214—H21J	108.1
C111—C19—C14	110.7 (11)	C216—C215—C214	109.8 (10)
C111—C19—H19A	109.5	C216—C215—C220	110.6 (11)
C14—C19—H19A	109.5	C214—C215—C220	108.4 (10)
C111—C19—H19B	109.5	C216—C215—H215	109.4
C14—C19—H19B	109.5	C214—C215—H215	109.4
H19A—C19—H19B	108.1	C220—C215—H215	109.4
C16—C110—C111	108.0 (11)	C217—C216—C215	108.5 (10)
C16—C110—H11E	110.1	C217—C216—H21K	110.0
C111—C110—H11E	110.1	C215—C216—H21K	110.0
C16—C110—H11F	110.1	C217—C216—H21L	110.0
C111—C110—H11F	110.1	C215—C216—H21L	110.0
H11E—C110—H11F	108.4	H21K—C216—H21L	108.4
C19—C111—C18	109.8 (10)	C216—C217—C221	110.8 (11)
C19—C111—C110	109.5 (11)	C216—C217—C218	109.3 (11)
C18—C111—C110	107.6 (11)	C221—C217—C218	109.1 (10)
C19—C111—H111	110.0	C216—C217—H217	109.2
C18—C111—H111	110.0	C221—C217—H217	109.2
C110—C111—H111	110.0	C218—C217—H217	109.2
N12—C112—C113	115.3 (9)	C213—C218—C217	109.9 (9)
N12—C112—H11G	108.5	C213—C218—H21M	109.7
C113—C112—H11G	108.5	C217—C218—H21M	109.7
N12—C112—H11H	108.5	C213—C218—H21N	109.7
C113—C112—H11H	108.5	C217—C218—H21N	109.7
H11G—C112—H11H	107.5	H21M—C218—H21N	108.2
C118—C113—C112	111.1 (10)	C213—C219—C222	108.9 (9)
C118—C113—C119	108.2 (10)	C213—C219—H21O	109.9
C112—C113—C119	108.8 (10)	C222—C219—H21O	109.9
C118—C113—C114	108.6 (10)	C213—C219—H21P	109.9
C112—C113—C114	110.8 (9)	C222—C219—H21P	109.9
C119—C113—C114	109.4 (9)	H21O—C219—H21P	108.3
C113—C114—C115	109.6 (10)	C222—C220—C215	109.1 (10)
C113—C114—H11I	109.8	C222—C220—H22C	109.9
C115—C114—H11I	109.8	C215—C220—H22C	109.9
C113—C114—H11J	109.8	C222—C220—H22D	109.9
C115—C114—H11J	109.8	C215—C220—H22D	109.9
H11I—C114—H11J	108.2	H22C—C220—H22D	108.3
C116—C115—C120	109.7 (11)	C222—C221—C217	108.7 (11)
C116—C115—C114	109.9 (10)	C222—C221—H22E	109.9
C120—C115—C114	109.8 (10)	C217—C221—H22E	109.9
C116—C115—H115	109.1	C222—C221—H22F	109.9
C120—C115—H115	109.1	C217—C221—H22F	109.9
C114—C115—H115	109.1	H22E—C221—H22F	108.3
C115—C116—C117	110.3 (11)	C220—C222—C221	112.0 (11)
C115—C116—H11K	109.6	C220—C222—C219	109.6 (10)
C117—C116—H11K	109.6	C221—C222—C219	108.0 (10)
C115—C116—H11L	109.6	C220—C222—H222	109.1
C117—C116—H11L	109.6	C221—C222—H222	109.1
H11K—C116—H11L	108.1	C219—C222—H222	109.1
C118—C117—C116	108.5 (11)	C1—N1—C3	119.7 (11)
C118—C117—C121	108.7 (12)	C1—N1—C4	122.7 (11)
C116—C117—C121	108.6 (11)	C3—N1—C4	117.6 (11)
C118—C117—H117	110.3	O1—C1—N1	125.5 (13)
C116—C117—H117	110.3	O1—C1—H1	117.2
C121—C117—H117	110.3	N1—C1—H1	117.2
C113—C118—C117	111.2 (10)	N1—C3—H3A	109.5
C113—C118—H11M	109.4	N1—C3—H3B	109.5
C117—C118—H11M	109.4	H3A—C3—H3B	109.5
C113—C118—H11N	109.4	N1—C3—H3C	109.5
C117—C118—H11N	109.4	H3A—C3—H3C	109.5
H11M—C118—H11N	108.0	H3B—C3—H3C	109.5
C122—C119—C113	111.3 (11)	N1—C4—H4A	109.5
C122—C119—H11O	109.4	N1—C4—H4B	109.5
C113—C119—H11O	109.4	H4A—C4—H4B	109.5
C122—C119—H11P	109.4	N1—C4—H4C	109.5
C113—C119—H11P	109.4	H4A—C4—H4C	109.5
H11O—C119—H11P	108.0	H4B—C4—H4C	109.5
C115—C120—C122	108.3 (10)	C2—N2—C5	119.3 (10)
C115—C120—H12C	110.0	C2—N2—C6	121.7 (10)
C122—C120—H12C	110.0	C5—N2—C6	119.0 (10)
C115—C120—H12D	110.0	O2—C2—N2	123.5 (11)
C122—C120—H12D	110.0	O2—C2—H2	118.2
H12C—C120—H12D	108.4	N2—C2—H2	118.2
C122—C121—C117	109.1 (10)	N2—C5—H5A	109.5
C122—C121—H12E	109.9	N2—C5—H5B	109.5
C117—C121—H12E	109.9	H5A—C5—H5B	109.5
C122—C121—H12F	109.9	N2—C5—H5C	109.5
C117—C121—H12F	109.9	H5A—C5—H5C	109.5
H12E—C121—H12F	108.3	H5B—C5—H5C	109.5
C119—C122—C121	109.3 (11)	N2—C6—H6A	109.5
C119—C122—C120	109.6 (10)	N2—C6—H6B	109.5
C121—C122—C120	110.4 (11)	H6A—C6—H6B	109.5
C119—C122—H122	109.2	N2—C6—H6C	109.5
C121—C122—H122	109.2	H6A—C6—H6C	109.5
C120—C122—H122	109.2	H6B—C6—H6C	109.5
			
N12—Pt1—N11—C11	−118.0 (8)	C115—C120—C122—C119	−59.8 (14)
Cl11—Pt1—N11—C11	64.4 (8)	C115—C120—C122—C121	60.7 (14)
N11—Pt1—N12—C112	122.5 (8)	Pt2—N21—C21—C22	162.7 (7)
Cl12—Pt1—N12—C112	−58.5 (7)	N21—C21—C22—C28	−62.7 (13)
N22—Pt2—N21—C21	123.0 (8)	N21—C21—C22—C23	177.4 (9)
Cl21—Pt2—N21—C21	−58.1 (8)	N21—C21—C22—C27	57.5 (12)
N21—Pt2—N22—C212	−114.7 (8)	C21—C22—C23—C24	178.9 (10)
Cl22—Pt2—N22—C212	68.2 (7)	C28—C22—C23—C24	57.5 (12)
Pt1—N11—C11—C12	−159.0 (8)	C27—C22—C23—C24	−59.3 (13)
N11—C11—C12—C18	−179.9 (9)	C22—C23—C24—C29	−58.8 (14)
N11—C11—C12—C17	−59.8 (13)	C22—C23—C24—C25	61.7 (13)
N11—C11—C12—C13	62.8 (13)	C29—C24—C25—C26	59.9 (14)
C11—C12—C13—C14	176.4 (11)	C23—C24—C25—C26	−61.6 (14)
C18—C12—C13—C14	59.6 (13)	C24—C25—C26—C210	−58.2 (14)
C17—C12—C13—C14	−58.9 (14)	C24—C25—C26—C27	59.8 (14)
C12—C13—C14—C15	59.4 (14)	C25—C26—C27—C22	−57.7 (14)
C12—C13—C14—C19	−60.0 (14)	C210—C26—C27—C22	61.8 (14)
C13—C14—C15—C16	−60.2 (14)	C21—C22—C27—C26	176.5 (10)
C19—C14—C15—C16	59.2 (14)	C28—C22—C27—C26	−61.0 (13)
C14—C15—C16—C110	−60.4 (14)	C23—C22—C27—C26	56.8 (13)
C14—C15—C16—C17	60.4 (14)	C21—C22—C28—C211	−177.8 (11)
C11—C12—C17—C16	−177.5 (10)	C23—C22—C28—C211	−58.1 (14)
C18—C12—C17—C16	−58.4 (13)	C27—C22—C28—C211	59.4 (13)
C13—C12—C17—C16	59.4 (13)	C23—C24—C29—C211	58.1 (15)
C110—C16—C17—C12	60.7 (14)	C25—C24—C29—C211	−61.5 (14)
C15—C16—C17—C12	−60.7 (13)	C25—C26—C210—C211	59.1 (14)
C11—C12—C18—C111	−177.0 (10)	C27—C26—C210—C211	−60.3 (14)
C17—C12—C18—C111	60.4 (13)	C22—C28—C211—C210	−59.5 (14)
C13—C12—C18—C111	−58.2 (12)	C22—C28—C211—C29	58.2 (15)
C15—C14—C19—C111	−61.1 (14)	C26—C210—C211—C28	59.4 (14)
C13—C14—C19—C111	59.1 (14)	C26—C210—C211—C29	−59.4 (14)
C15—C16—C110—C111	59.1 (14)	C24—C29—C211—C28	−57.2 (15)
C17—C16—C110—C111	−61.2 (14)	C24—C29—C211—C210	61.1 (15)
C14—C19—C111—C18	−57.2 (14)	Pt2—N22—C212—C213	−160.5 (7)
C14—C19—C111—C110	60.8 (14)	N22—C212—C213—C214	179.9 (9)
C12—C18—C111—C19	58.2 (14)	N22—C212—C213—C218	60.4 (13)
C12—C18—C111—C110	−60.9 (13)	N22—C212—C213—C219	−62.5 (13)
C16—C110—C111—C19	−59.2 (14)	C212—C213—C214—C215	−179.3 (9)
C16—C110—C111—C18	60.1 (14)	C218—C213—C214—C215	−58.0 (12)
Pt1—N12—C112—C113	163.9 (7)	C219—C213—C214—C215	60.9 (12)
N12—C112—C113—C118	−62.2 (12)	C213—C214—C215—C216	59.3 (12)
N12—C112—C113—C119	178.8 (8)	C213—C214—C215—C220	−61.6 (12)
N12—C112—C113—C114	58.6 (12)	C214—C215—C216—C217	−60.1 (13)
C118—C113—C114—C115	−59.7 (12)	C220—C215—C216—C217	59.4 (13)
C112—C113—C114—C115	178.1 (10)	C215—C216—C217—C221	−59.4 (14)
C119—C113—C114—C115	58.2 (13)	C215—C216—C217—C218	60.9 (13)
C113—C114—C115—C116	60.4 (13)	C212—C213—C218—C217	176.0 (10)
C113—C114—C115—C120	−60.4 (13)	C214—C213—C218—C217	58.5 (12)
C120—C115—C116—C117	61.2 (14)	C219—C213—C218—C217	−59.8 (12)
C114—C115—C116—C117	−59.7 (14)	C216—C217—C218—C213	−60.7 (12)
C115—C116—C117—C118	58.3 (15)	C221—C217—C218—C213	60.6 (13)
C115—C116—C117—C121	−59.7 (15)	C212—C213—C219—C222	−175.1 (10)
C112—C113—C118—C117	−177.8 (10)	C214—C213—C219—C222	−58.8 (12)
C119—C113—C118—C117	−58.5 (13)	C218—C213—C219—C222	60.2 (12)
C114—C113—C118—C117	60.1 (13)	C216—C215—C220—C222	−59.0 (14)
C116—C117—C118—C113	−58.9 (14)	C214—C215—C220—C222	61.3 (13)
C121—C117—C118—C113	59.0 (13)	C216—C217—C221—C222	58.2 (14)
C118—C113—C119—C122	59.5 (12)	C218—C217—C221—C222	−62.2 (13)
C112—C113—C119—C122	−179.7 (9)	C215—C220—C222—C221	58.5 (14)
C114—C113—C119—C122	−58.6 (13)	C215—C220—C222—C219	−61.3 (13)
C116—C115—C120—C122	−60.4 (13)	C217—C221—C222—C220	−58.2 (14)
C114—C115—C120—C122	60.5 (13)	C217—C221—C222—C219	62.6 (13)
C118—C117—C121—C122	−59.1 (13)	C213—C219—C222—C220	60.5 (13)
C116—C117—C121—C122	58.8 (15)	C213—C219—C222—C221	−61.8 (13)
C113—C119—C122—C121	−61.4 (13)	C3—N1—C1—O1	−178.5 (14)
C113—C119—C122—C120	59.7 (13)	C4—N1—C1—O1	3(2)
C117—C121—C122—C119	60.3 (14)	C5—N2—C2—O2	176.5 (11)
C117—C121—C122—C120	−60.4 (14)	C6—N2—C2—O2	−1.7 (18)

### Hydrogen-bond geometry (Å, °)

**Table:**

*D*—H···*A*	*D*—H	H···*A*	*D*···*A*	*D*—H···*A*
N11—H11A···O2	0.92	1.95	2.863 (13)	170
N11—H11B···Cl12^i^	0.92	2.64	3.409 (10)	141
N12—H12A···Cl11^i^	0.92	2.63	3.299 (9)	131
N12—H12B···O2	0.92	1.93	2.843 (11)	175
N21—H21A···Cl22^ii^	0.92	2.55	3.238 (10)	132
N21—H21B···O1	0.92	1.91	2.821 (12)	173
N22—H22A···O1	0.92	1.94	2.843 (11)	165
N22—H22B···Cl21^ii^	0.92	2.64	3.354 (10)	136

Symmetry codes: (i) −*x*+2, −*y*, −*z*+2; (ii) −*x*+1, −*y*+1, −*z*+2.
